# Increased Serum Concentrations of High Mobility Group Box 1 (HMGB1) Protein in Children with Autism Spectrum Disorder

**DOI:** 10.3390/children8060478

**Published:** 2021-06-05

**Authors:** Gerasimos Makris, Giorgos Chouliaras, Filia Apostolakou, Charalabos Papageorgiou, George P. Chrousos, Ioannis Papassotiriou, Panagiota Pervanidou

**Affiliations:** 1 Laboratory of Developmental Psychophysiology and Stress Research, Unit of Developmental and Behavioral Pediatrics, First Department of Pediatrics, “Aghia Sophia” Children’s Hospital, School of Medicine, National and Kapodistrian University of Athens, 11527 Athens, Greece; georgehouliaras@msn.com (G.C.); chrousos@gmail.com (G.P.C.); ppervanid@med.uoa.gr (P.P.); 2 Department of Clinical Biochemistry, “Aghia Sophia” Children’s Hospital, 11527 Athens, Greece; fapostolakou@yahoo.gr (F.A.); biochem@paidon-agiasofia.gr (I.P.); 3 First Department of Psychiatry, “Eginition” University Hospital, School of Medicine, National and Kapodistrian University of Athens, 11528 Athens, Greece; chpapag@med.uoa.gr

**Keywords:** high mobility group box 1, neurodevelopmental disorders, autism spectrum disorder, immune dysfunction, systemizing quotient, autism spectrum quotient

## Abstract

High mobility group box 1 protein (HMGB1) has been suggested to be involved in the immune dysfunction and inflammation reported in autism spectrum disorder (ASD). We aimed to assess HMGB1 serum concentrations (SCs) in high-functioning ASD children compared to typically developing (TD) controls and to explore their associations with the autism spectrum quotient (AQ), the empathy quotient (EQ), and the systemizing quotient (SQ). The study involved 42 ASD children and 38 TD children, all-male, aged between 6.1 and 13.3 years old. HMGB1 SCs were measured by enzyme-linked immunosorbent assay (ELISA). Groups were comparable regarding age, general IQ, birth weight, and maternal age at birth. ASD children showed significantly higher HMGB1 SCs compared to TD children (1.25 ± 0.84 ng/mL versus 1.13 ± 0.79 ng/mL, respectively, *p* = 0.039). The Spearman’s rho revealed that HMGB1 SCs were positively correlated with the AQ attention to detail subscale (rs = 0.46, *p* = 0.045) and with the SQ total score (rs = 0.42, *p* = 0.04) in the ASD group. These results show that HMGB1 serum concentrations are altered in ASD children, and suggest that inflammatory processes mediated by HMGB1 may be associated with specific cognitive features observed in ASD.

## 1. Introduction

Autism spectrum disorder (ASD) is a neurodevelopmental disorder, which occurs in early childhood and is characterized by impairments in social communication and by restricted, repetitive, and stereotyped patterns of behavior [1]. To date, the exact etiology and underlying neuropathology of ASD remain largely unknown, although it is likely to result from a complex combination of environmental, neurological, immunological, genetic, and epigenetic factors [2,3]. Among several biological processes, it has been proposed that immune dysfunction and inflammation may play a key role in the pathophysiology of ASD [4]. Altered immune responses have been reported in ASD ranging from alterations of immune markers in the periphery to increased microglia activation in the central nervous system (CNS), all of them leading to a chronic state of low-grade inflammation in the CNS [4,5,6]. Several studies have shown peripheral immune abnormalities in patients with ASD, including abnormal or skewed T helper cell cytokine profiles [3], an imbalance of serum immunoglobulin levels [7], NK cell activation [8], increased monocyte responses [9], and increased levels of complement components [10]. Moreover, several studies have demonstrated increased levels of plasma/serum pro-inflammatory cytokines in ASD, such as interleukin (IL)-1β, IL-6, IL-8, IL-12p40, IL-12, interferon-γ (INF-γ) or a decreased production of cytokines that negatively regulate inflammation, such as TGFβ1 [4,11,12,13]. Finally, the alarmin family, which comprises a heterogeneous group of proteins released in the extracellular space as a consequence of cell damage or inflammation, has been implicated in the pathogenesis of ASD [14,15]. Precisely, IL-33, HMGB1, heat-shock protein (HSP), and S100 protein could be suitable as biomarkers of inflammation in ASD [4].

High mobility group box 1 protein (HMGB1) (also called HMG1; HMG-1; HMG 1; amphoterin; p30) is an evolutionarily highly conserved intracellular protein, widely expressed in all tissues of vertebrates [16]. HMGB1 is the most abundant member of the HMGB protein family comprised of four categories of HMGB, from 1 to 4 [14]. It is the most mobile protein in the nucleus and can be found in the cytosol, the cellular membrane, and the extracellular space [17]. Nuclear HMGB1 exerts DNA binding, with structure-specificity, but not sequence-specificity, and bending activities regulating DNA replication, repair, recombination, transcription, and genomic stability [17]. The extracellular HMGB1 can be actively secreted under inflammatory conditions as an alarmin or late pro-inflammatory cytokine by different kinds of cells including monocytes, tissue macrophages, astrocytes, microglia, and neurons [18]. Additionally, HMGB1 can be passively released from dead, dying, or injured cells [17]. Thus, HMGB1 can be either an early inflammatory index, in the case of passive release, or a late mediator, in the case of active secretion [16]. Once secreted, HMGB1 takes part in several processes such as inflammation, immunity, migration, invasion, proliferation, differentiation, antimicrobial defense, and tissue regeneration [17]. Toll-like receptor 4 (TLR4), Toll-like receptor 9, and receptor for advanced glycation end products (RAGE) are the dominant HMGB1-receptors, through which it exerts its pro-inflammatory activity [18]. Moreover, HMGB1 is able to cross the blood-brain barrier and therefore the brain cells may be exposed to HMGB1 released both in the brain and in the periphery [19]. Regarding the CNS, HMGB1 either at the cell surface of neurons or in the extracellular matrix has a role in the promotion of neurite outgrowth and cell migration or mediates neuroinflammation after injury [19].

In view of the above, HMGB1 is a candidate biomarker that may be involved in altered molecular pathways leading to the immune dysfunction reported in individuals with ASD. Previous studies have reported higher HMGB1 serum or plasma concentrations mainly in low-functioning ASD individuals comprising samples of young adults (18 to 44 years old) [20], male children (mean age 10.6 years) [21], and ASD individuals aged 2–22 years [22], compared with age- and gender-matched healthy controls. The objective of the current cross-sectional study was to assess the concentrations of the HMGB1 in serum samples of school-aged, male children diagnosed with high-functioning ASD (i.e., children of normal intelligence) compared to typically developing (TD) controls. We hypothesized that children with high-functioning ASD would have higher HMGB1 serum levels compared to TD children. Moreover, we intended to contribute to existing evidence regarding the role of immune system alterations in the core symptoms of ASD. HMGB1 serum concentrations have been found to positively correlate with deficits in social interaction as assessed with the Autism Diagnostic Interview-Revised (ADI-R) in young adults with ASD [20]. In the current study, we explore for the first time the relations of HMGB1 serum levels to the autism spectrum quotient, and to the empathizing (empathy quotient) and systemizing abilities (systemizing quotient) that have been considered accountable for social deficits and non-social phenotypic characteristics of ASD.

## 2. Materials and Methods

### 2.1. Study Design and Participants

The current study was a cross-sectional case-control survey conducted between December 2016 and November 2018. A total of 80 male schoolchildren, aged between 6.1 and 13.3 years old, were enrolled in the study. Children were distributed into two groups: Forty-two children were clinically diagnosed with ASD and 38 TD children comprised the comparison group. All children were of normal intelligence (IQ > 70). The descriptive characteristics of the study population are presented in Table 1.

The participants of the clinical group derived from the referrals of the Outpatient Unit of Developmental and Behavioral Pediatrics, First Department of Pediatrics, “Aghia Sophia” Children’s Hospital, School of Medicine, National and Kapodistrian University of Athens. The children of typical development were recruited from the community after a public call through printed and electronic newspapers. All children participated in the study with their parent’s written informed consent. All procedures were in accordance with the Declaration of Helsinki and approved by both the Scientific and the Ethics Committee of the Children’s Hospital.

### 2.2. Assessments

#### 2.2.1. Clinical Diagnoses and Exclusion Criteria

A full clinical examination was performed in all children, including the group of TD children, and a clinical interview with their caregivers was conducted. The clinical diagnoses were established by a developmental-behavioral pediatrician with extensive clinical and research experience and according to standard criteria based on the Diagnostic and Statistical Manual of Mental Disorders (DSM), 5th edition (American Psychiatric Association, 2013). All children diagnosed with ASD had a multidisciplinary diagnostic assessment and several previous assessments in the context of annual follow-up visits at the Unit of Developmental and Behavioral Pediatrics. All children comprising the ASD group were diagnosed between 3 and 5 years of age. At the time of the study, all children were undergoing non-pharmacological interventions based on behavioral approaches. For children diagnosed before 2013 according to the DSM-IV/TR criteria, only those fulfilling also the DSM-5 criteria were included in the study. Additionally, the co-occurrence of ASD and ADHD was taken into account as an exclusion criterion for the clinical groups. The Child Behavior Checklist/6–18 and the Teacher’s Report Form of the Achenbach System of Empirically Based Assessment were administered to all participants in order to screen for other behavioral and emotional comorbid conditions [23,24]. All participants who exhibited at least one comorbid condition at the clinical level were excluded from the study. In addition, the children’s intelligence quotient (IQ) was assessed using the Greek version of the Wechsler Intelligence Scale for Children- Third Edition (WISC-III) [25]. By inclusion criteria, only children with performances within the normal range entered the study.

Individuals with any infectious disease in the last four weeks, an IQ lower than 70, genetic syndromes or chromosomal abnormalities, comorbid autoimmune, endocrine, metabolic or other chronic disorders or conditions, comorbid neurological or other psychiatric diseases were excluded from the study. Additionally, subjects who received any kind of medication were excluded. Furthermore, extreme prematurity (<30 weeks) was an additional exclusion criterion during the sampling procedure. Finally, overweight and obese subjects [Body mass index (BMI) above the 85th percentile for age and sex] were excluded from the study. By the end of the recruitment process, a total of 90 subjects, 50 ASD and 40 TD, met the inclusion criteria and agreed to participate in the study. However, at some point in the study, 2 ASD children and 1 TD child were diagnosed with endocrine disorders, 6 ASD children were diagnosed with psychiatric co-morbidities, and 1 TD child was diagnosed with attention deficit hyperactivity disorder (ADHD). Thus, their participation was discontinued and they were not included in the analysis.

#### 2.2.2. Psychometric Questionnaires

The instruments administered in the current study comprised the Greek adaptations of i. the empathy quotient (EQ) and systemizing quotient (SQ)—children’s version, combined into one questionnaire (EQ-SQ), and ii. the autism spectrum quotient (AQ)—children’s version (AQ-Child). Both questionnaires were translated into the Greek language following the WHO guidelines for translation and adaptation of instruments, with the kind permission of the authors: Autism Research Centre website (http://www.autismresearchcentre.com/).

The EQ and SQ are based on Baron-Cohen’s empathizing-systemizing theory and the extreme male brain theory in ASD [26]. The EQ-SQ questionnaire includes 55 items (27 EQ and 28 SQ questions), which are designed to assess the empathizing (i.e., the drive to identify another person’s emotions and thoughts and to respond to these with an appropriate emotion) and the systemizing (i.e., the drive to analyze, explore and construct a system) quotient of children between 5 and 12 years old, reported by parents/caregivers [27]. Parents/caregivers indicate how strongly they agree with each statement by choosing one of the options: “definitely agree”, “slightly agree”, “slightly disagree” or “definitely disagree”. A “slightly agree” response scores one point and a “definitely agree” scores two points. The maximum attainable score for the EQ is 54 and for the SQ is 56. Two scores were derived: i. EQ total and ii. SQ total. The original tools in English have shown good test-retest reliability and high internal consistency [27]. In general terms, children with ASD score significantly lower on the EQ and significantly higher on the SQ than TD children [27]. Moreover, low empathizing skills have been associated with the social deficits that ASD individuals experience, and the high systemizing skills have been associated with the restricted, repetitive patterns of behavior, interests, or activities in this population [28].

The AQ-child includes 50 statements, which are designed to assess the autistic traits of children between 4 and 11 years old, reported by parents/caregivers [29]. The statements assess five areas associated with ASD (i.e., communication, social skills, attention switching, attention to detail, and imagination). Each domain is represented by ten items. Parents/caregivers rate to what extent they agree with each statement about their child by choosing one of the options: “definitely agree”, “slightly agree”, “slightly disagree” or “definitely disagree”. The response scale is treated as a 4-point Likert scale from zero to three. The maximum attainable score is 150. The higher score represents the greater autistic traits. Six scores were derived: i. AQ communication; ii. AQ social skills; iii. AQ attention switching; iv. AQ attention to detail; v. AQ imagination; vi. AQ total.

### 2.3. Serum Samples Collection and Analysis

Blood sampling was performed between 8:00 and 10:00 a.m. after an overnight fast (≥8 h). Venous blood samples were drawn by the venipuncture technique into vacutainer tubes without anticoagulants and were allowed to clot for 30 min. The samples were centrifuged at 1000× *g* for 15 min and aliquots were stored at −80 °C until immediately before analysis.

All sample analyses were performed at the Department of Clinical Biochemistry, “Aghia Sophia” Children’s Hospital, Athens, Greece. Laboratory personnel were blinded to the case or control status. The determination of HMGB1 serum levels was performed with an enzyme-linked immunosorbent assay (ELISA) kit according to the manufacturer’s instructions (Arigo Biolaboratories Co., Hsinchu City, Taiwan, ROC). This assay recognizes natural and recombinant total human/Mouse/Rat HMGB1. The inter- and intra-assay coefficients of variation were 7.6% and 5.2% respectively. HMGB1 levels were expressed in ng/mL and the limit of detection (LOD) was 0.4 ng/mL. All samples were analyzed in duplicate in the same assay.

### 2.4. Statistical Analysis

Continuous variables are presented as mean ± standard deviation (SD), median and interquartile range (IQR). Categorical data are displayed as absolute (*n*) and relative frequencies (%). Normality distribution was assessed for all the quantitative variables with the Shapiro-Wilk test. With the exception of age and HMGB1, the normality of the data was not rejected. Log-transformation of age improved approximation of the normal distribution (not rejected). In contrast, log-HMGB1 did not follow the normal distribution; nevertheless, it showed less skewness compared to raw data and therefore log-transformed values were used for the parametric linear regression analysis. Comparisons of continuous data between two groups were performed with the Student’s *t*-test or the Mann-Whitney U test; associations between categorical variables were evaluated by the Fisher’s exact test. Correlations between continuous parameters were examined by Pearson’s correlation coefficient (Pearson’s r) or Spearman’s correlation coefficient (Spearman’s rho). Bivariate analyses were performed to assess the difference of HMGB1 serum levels between ASD and TD groups, as well as correlations between HMGB1 levels and demographic, gestational, IQ, and psychometric variables. A stepwise backward linear regression approach was utilized to assess the relations between log-HMGB1 and explanatory covariates. Results were reported as β-coefficients, along with 95% confidence interval (ci) and *p*-values. The level of statistical significance was set to 0.05. All analyses were performed using Stata 11 MP statistical software (StataCorp, College Station, TX, USA).

## 3. Results

All subjects were comparable regarding age, general IQ, birth weight, and maternal age at birth. Children in the ASD group had significantly lower verbal IQ compared to TD children. As expected, significantly higher scores for ASD than TD children were shown for the total AQ and on all AQ subscales, except for the AQ attention to detail subscale. Moreover, EQ was significantly lower in the ASD group compared to TD children. Nevertheless, no significant difference was shown regarding SQ between the two groups (Table 1).

**Figure 1:**
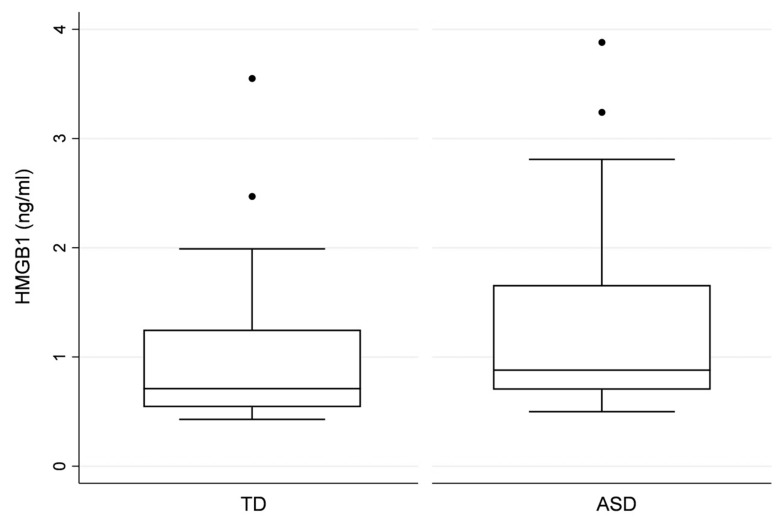
Distribution of HMBG1 serum concentrations in ASD and TD children.

Concerning HMGB1 serum concentrations (SC), ASD children showed significantly higher HMGB1 serum levels compared to the group comprised of TD children. Nevertheless, the HMGB1 detection rate (DR) (i.e., the number/proportion of individuals with HMGB1 serum concentrations above the LOD) did not differ between the two groups (Table 2). Additionally, there were no differences regarding the AQ, SQ, and EQ variables between the subjects with HMGB1 serum concentrations above the LOD and subjects with no detectable HMGB1 levels. The distribution of HMBG1 serum concentrations in ASD and TD children is presented as box plots in Figure 1.

**Figure 2:**
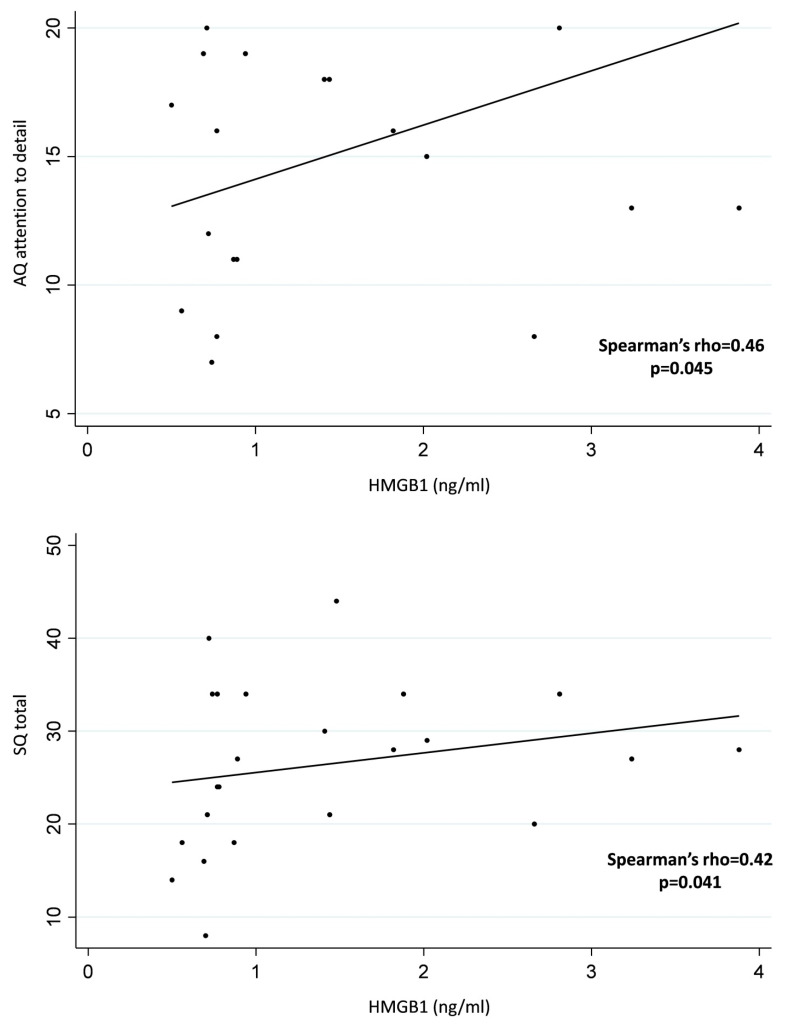
Positive associations between HMGB1 serum concentrations and AQ attention to detail and SQ total scores in ASD children.

No associations between HMGB1 SCs and age, general IQ, verbal IQ, birth weight, and maternal age at birth were found. The analysis regarding the associations between HMGB1 SCs and AQ, EQ, and SQ variables showed that HMGB1 SCs were positively correlated with the AQ attention to detail and with the SQ total score in the ASD group. Table 3 illustrates correlations between HMGB1 SCs and AQ, EQ, SQ, and IQ variables in the ASD group (Table 3). Positive associations between HMGB1 serum concentrations and AQ attention to detail and SQ total scores in the ASD group are presented in Figure 2. Overall, the multivariate analysis showed that log-HMGB1 was positively associated to AQ attention to detail (β-coefficient: 0.08, 95% ci: 0.02, 0.13, *p* = 0.006) and inversely related to SQ total (β-coefficient: −0.058, 95% ci: −0.09, −0.01, *p* = 0.009).

## 4. Discussion

In the present study, we have shown that HMGB1 serum concentrations in children with ASD are significantly higher than those of TD children. Additionally, we found that HMGB1 serum concentrations are positively correlated with the AQ attention to detail and the SQ total score in the ASD group. To date, only a few studies have investigated HMGB1 serum concentrations in individuals with ASD, and the majority of them concern young adult patients. Importantly, unlike the previous studies investigating HMGB1 levels in ASD individuals, the current clinical sample comprised exclusively unmedicated children diagnosed with high-functioning ASD.

Our findings regarding HMGB1 levels are in line with previous studies that assessed HMGB1 serum or plasma levels in individuals with ASD. Precisely, higher HMGB1 serum concentrations have been firstly reported in a sample of young adults aged from 18 to 44 years with low-functioning ASD compared with age- and gender-matched healthy controls. Additionally, HMGB1 SCs were found to positively correlate with deficits in social interaction as assessed with the Autism Diagnostic Interview-Revised (ADI-R) [20]. Similar findings have been observed in plasma samples of male children (mean age 10.6 years) diagnosed with ASD in comparison to TD children [21]. Moreover, research has shown that HMGB1 plasma levels are related to a low concentration of epidermal growth factor (EGF) and higher EGF receptor (EGFR) levels in ASD individuals, suggesting that both EGF and EGFR abnormal levels found in ASD persons may be associated with inflammation and generally increased neuroimmune activity [21,30]. Furthermore, in a study comprising low-functioning ASD individuals aged 2–22 years, plasma HMGB1 levels were found significantly higher in the ASD group than in controls [22]. Additionally, elevated HMGB1 plasma and fecal levels have been associated with higher severity of gastrointestinal (GI) problems in children and young individuals with ASD [22,31]. Recent findings of note have shown that HMGB1 may play a role in the stress-induced sensitization of innate immune cells and subsequent neuroinflammation [32]. Although the mechanism of stress-induced increase of HMGB1 is largely unknown, it has been suggested that glucocorticoids may function as an alarmin by inducing HMGB1 [33,34]. Nevertheless, to date, there is no evidence regarding the association between stress and HMGB1 in ASD individuals.

HMGB1 receptors may be involved in the pathophysiological mechanisms of ASD. Regarding TLR signaling, peripheral blood monocyte cultures from children with ASD have been found more responsive to TLR ligands compared to controls, indicating an underlying dysfunction in monocyte pathogen recognition and/or TLR signaling pathways [9,35]. Moreover, increased TLR4 expression has been found on T cells isolated from ASD children compared to TD controls [36]. The activation of TLR4 signaling leads to up-regulation of NADPH oxidase (NOX-2) dependent reactive oxygen species (ROS) generation by immune cells, which may be a key mechanism for causing neuroinflammation in individuals with ASD [36]. Interestingly, higher circulating levels and increased expression of HMGB1 and TLR4 in epileptogenic tissue have been associated with increased risk and severity of epilepsy [37,38,39]. The HMGB1-TLR4 interaction mediates changes in voltage- and ligand-gated ion channels resulting in neuronal hyperexcitability [40]. In addition, it induces transcriptional changes in genes related to neurotransmission and synaptic plasticity contributing to perpetual inflammation and chronically lower seizure thresholds [40]. Therefore, it is possible that the interaction of HMGB1 with TLR4 constitutes a potential link between ASD and epilepsy [41,42].

Recently, it has been shown in animal models that HMGB1 may up-regulate the expression of TLR4 in the plasma membrane and also may increase RAGE expression in both the cytoplasm and plasma membrane [43]. Plasma RAGE has been found significantly higher in male children with ASD (mean age 10.6 years), particularly those with GI disease, compared to TD children, suggesting that the HMGB1/RAGE pathway may be associated with inflammation in ASD individuals [44]. On the other hand, in a study comprising 18 ASD and 18 age- and gender-matched TD subjects, aged 15–42 years, reduced plasma levels of endogenous secretory RAGE (esRAGE) coupled with elevated S100A9 were found in ASD individuals as compared to controls [45]. However, no significant correlation was found between S100A9 and esRAGE, suggesting that RAGE levels may be reduced in ASD because of its binding to ligands besides S100A9, such as HMGB1 among others (i.e., advanced glycation end products, lipopolysaccharides, amyloid-beta peptide) [45]. Moreover, it has been suggested that RAGE in the blood-brain barrier (BBB) endothelial cells may be the transporter of oxytocin (OXT) from the periphery into the brain [46]. Additionally, it is highly possible that esRAGE, which can be transported into the brain through the BBB, serves as an OXT binding protein contributing to OXT transfer into the brain, hence resulting in the regulation of brain OXT levels [47,48]. To date, it is well established that the neuropeptide OXT is critically involved in ASD pathophysiology due to its effects on emotional and social behavior [49,50,51]. Therefore, we suppose that elevated HMGB1 levels may contribute to some extent to the dysregulation of the RAGE-mediated OXT transport from the periphery to the brain, associated with social deficits characterizing ASD.

An intriguing finding of the present study is that higher HMGB1 serum levels in the ASD group were correlated with the attention to detail subscale of the AQ and with higher SQ. The attention to detail subscale captures the tendency to process sensory input in a detailed-focused or piecemeal way at the expense of more integrative perceptions [29]. In the same line, systemizing refers to the drive to analyze, explore and construct a system and thus recognize repeating patterns or regularities in stimuli [27,52]. Although attention occurs at an earlier level of cognition than systemizing, it has been proposed that attention to detail and systemizing are closely intertwined in the sense that the former is in the service of the latter [52]. Interestingly, the autistic trait of attention to detail has been associated with weaker temporal recalibration (i.e., decreased ability to perceptually realign physically asynchronous stimuli) [53,54]. To date, it is well documented that increased attention to detail and high systemizing abilities characterize the cognitive style broadly observed in ASD [28]. In addition, such cognitive style may account to a certain extent for the non-social phenotypic characteristics of ASD, such as the restricted interests and repetitive behaviors [28]. In fact, increased inflammation, such as microglia activation and elevated cytokine levels, has been associated with cognitive alterations across several psychiatric conditions [55]. The associations of higher HMGB1 levels with increased attention to detail and higher systemizing demonstrated in the current study are indicative of the fact that inflammatory processes mediated by HMGB1 may play a role in the disruption of neurobiological mechanisms regulating cognitive processes in ASD [56].

In view of the above, future studies may investigate the role of HMGB1 as a potential biomarker and therapeutic target in ASD individuals. To date, preclinical HMGB1-targeted therapy studies have demonstrated the efficacy of HMGB1 inhibitors in reducing inflammation in a broad set of infectious and sterile inflammatory conditions [16,57,58]. Nevertheless, these preclinical studies need to be translated to a clinical setting. More research is needed to clarify the role of HMGB1 in the pathophysiology of ASD and to cast light on the specific molecular mechanisms of the involvement of HMGB1 in ASD.

Several limitations should be considered in weighing the results of this study. First and foremost the cross-sectional design cannot infer causality of the observed associations. Additionally, the relatively small sample size of our population and the fact that it comprised only male individuals limit the generalizability of our results. In addition, the differences between groups and the associations observed in the current study were relatively modest, which may partially be related to the fact that the clinical group comprised exclusively high-functioning ASD children (i.e., children of normal IQ). Supposedly, HMGB1 serum levels may differ between ASD children with and without accompanying intellectual impairment. Nevertheless, in the current study, we did not find any associations between HMGB1 serum concentrations and the IQ variables. Similarly, a previous study that explored the associations of several inflammatory biomarkers, including HMGB1, with intellectual disability in children with Down syndrome did not find significant statistical correlations between serum HMGB1 levels and IQ [59]. A major limitation is that serum HMGB1 levels assessed by standard ELISA methods in the current study concern the fully reduced and non-acetylated isoform of the molecule [16]. However, the redox states of extracellular HMGB1 determine its biological functions. There are only a few laboratories worldwide capable of measuring HMGB1 isoforms through mass spectrometry, including the disulfide and the fully oxidized HMGB1 [16]. Briefly, the fully reduced HMGB1 triggers inflammatory responses via RAGE, the disulfide isoform via TLR4, and the fully oxidized HMGB1 has no chemokine or cytokine activities [60]. Different HMGB1 isoforms have not yet been assessed in the investigation of the association between immune dysregulation and ASD. Moreover, HMGB1 which is also present in microvesicles can escape measurement by ELISA unless lysed [61]. However, we used undiluted samples given that ASD individuals exhibit mainly low-grade inflammation accompanied by low-grade oxidative stress [13]. Thus, we did not expect high levels of HMGB1, such as observed in high aseptic inflammation of exercise [62] or in sepsis [63]. In addition, serum samples were not treated with perchloric acid purification prior to ELISA [64]. Therefore, we cannot rule out the possibility that various molecules, which have been shown to complex with HMGB1, may have decreased the ELISA sensitivity, at least regarding the masked forms of HMGB1 [64]. Finally, the soluble form of RAGE, which may capture and eliminate circulating HMGB1 in humans, and white blood cell counts have been independently associated with HMGB1 levels in the general population and may consist of possible confounding factors in the current study [65].

## 5. Conclusions

The results of this study add to the potential role of inflammatory processes in the pathophysiology of ASD, with special emphasis on HMGB1. Moreover, this study provides evidence of an association between raised levels of HMGB1 and attention to detail and systemizing in unmedicated, high-functioning ASD children, suggesting that inflammatory processes mediated by HMGB1 may play a role in the disruption of neurobiological mechanisms regulating cognitive processes in ASD. However, the physiological mechanisms of the observed associations remain largely unknown. Additionally, our results may not allow concrete conclusions regarding the extent to which HMGB1 mediates pathophysiological processes in ASD. Additionally, serum HMGB1 increases may affect also other domains that we have not included in the current study design. Yet, comprehensive evidence in children is limited, highlighting the need for in-depth research towards the understanding of possible mechanisms linking HMGB1 with the core features of ASD. Nevertheless, our findings support the hypothesis that HMGB1 could be a reliable inflammatory marker, explaining the link between inflammatory processes and several autistic traits, and therefore a possible therapeutic target in this neurodevelopmental disorder.

## Figures and Tables

**Table 1:** Descriptive characteristics ^a^ of the study population.

	TD ^b^ (*n* = 38)	ASD ^c^ (*n* = 42)	Total (*n* = 80)
Age (years)	9.1 ± 1.7, 8.8 (7.8–10.3)	8.3 ± 1.6, 7.7 (7.0–9.8) *p =* 0.05	8.7 ± 1.7, 8.7 (7.3–9.8)
Sex, males, *n* (%)	38 (100%)	42 (100%)	80 (100%)
General IQ	112.7 ± 13.8, 116.0 (105.0–122.0)	107.6 ± 16.9, 108.0 (95.0–121.0) *p =* 0.17	110.0 ± 15.6, 111.0 (100.0–121.5)
Verbal IQ	107.9 ± 16.0, 108.0 (96.0–117.0)	98.8 ± 18.2, 99.0 (86.0–114.5) ***p =* 0.03**	103.0 ± 17.7, 103.5 (91.0–115.5)
Birth weight (g)	3081 ± 653, 3125 (2740–3400)	3213 ± 440, 3200 (2900–3500) *p =* 0.35	3156.1 ± 541.4, 3200 (2845–3485)
Maternal age at birth (years)	32.2 ± 4.7, 32.0 (29.5–35.5)	33.6 ± 5.0, 33.0 (29.0–38.0) *p =* 0.28	32.9 ± 4.9, 32.0 (29.0–36.0)
AQ ^d^ Communication	6.5 ± 4.2, 7.0 (3.5–9.0)	12.5 ± 4.2, 12.5 (9.8–15.3) ***p* < 0.001**	9.5 ± 5.1, 9.0 (6.0–13.0)
AQ Social skills	7.2 ± 4.6, 8.0 (3.0–9.5)	11.0 ± 3.9, 11.0 (8.8–13.0) ***p =* 0.001**	9.1 ± 4.6, 9.0 (7.0–12.0)
AQ Attention switching	10.3 ± 3.9, 11.0 (6.5–13.0)	13.6 ± 4.3, 13.0 (10.0–18.0) ***p =* 0.002**	12.0 ± 4.4, 11.0 (9.0–15.0)
AQ Attention to detail	15.1 ± 5.1, 15.0 (12.0–17.0)	15.4 ± 5.1, 15.0 (11.8–20.0) *p =* 0.47	15.2 ± 5.0, 15.0 (12.0–18.0)
AQ Imagination	9.9 ± 3.7, 11.0 (10.8–15.3)	12.3 ± 3.8, 12.0 (29.5–35.5) ***p =* 0.009**	11.1 ± 3.9, 11.0 (9.0–14.0)
AQ Total	49.0 ± 11.5, 50.0 (44.0–55.0)	64.7 ± 13.3, 64.0 (56.0–73.5) ***p* < 0.001**	57.0 ± 14.7, 57.0 (46.0–65.0)
EQ ^e^ Total	36.8 ± 8.1, 36.0 (33.5–42.0)	30.2 ± 7.3, 29.0 (24.8–35.5) ***p* < 0.001**	33.5 ± 8.3, 34.0 (27.0–40.0)
SQ ^f^ Total	29.0 ± 7.3, 28.0 (23.0–34.5)	26.6 ± 6.7, 27.5 (20.8–32.3) *p =* 0.21	27.8 ± 7.0, 28.0 (22.0–34.0)

^a^ Data are presented as mean ± standard deviation, median (interquartile range) for continuous variables. Categorical outcomes are presented as absolute and relative frequencies, *n* (%). *p*-values refer to comparisons between TD and ASD groups (Student’s t-test). Statistically significant associations are shown in bold (*p* < 0.05); ^b^ TD: Typical development; ^c^ ASD: Autism Spectrum Disorder; ^d^ AQ: Autism spectrum quotient; ^e^ EQ: Empathy quotient; ^f^ SQ: Systemizing quotient.

**Table 2:** Detection rates (DRs) ^a^ and serum concentrations (SCs) ^b^ of HMGB1 in the study population.

	TD ^c^	ASD ^d^	Total
DR	30 (78.9%)	34 (81.0%) *p* = 0.99	64 (80.0%)
SC	0.99 ± 0.71, 0.71 (0.54, 1.25)	1.25 ± 0.84, 0.88 (0.70, 1.66) ***p* = 0.039**	1.13 ± 0.79, 0.80 (0.62, 1.44)

^a^ Detection rate (DR): the absolute number and proportion of individuals above the limit of detection (LOD). Comparisons for DRs and respective *p*-values were performed between the ASD and the TD groups (Fisher’s exact test); ^b^ SC: serum concentrations in nanograms per milliliter (ng/mL), presented as mean ± standard deviation, median (interquartile range). Comparisons for SCs and respective *p*-values were performed between the ASD and the TD groups (Mann-Whitney U test). Statistically significant associations are shown in bold (*p* < 0.05); ^c^ TD: Typical development; ^d^ ASD: Autism Spectrum Disorder.

**Table 3:** Correlations ^a^ between HMGB1 serum concentrations and AQ ^b^, EQ ^c^, SQ ^d^, and IQ scores in the total population and the ASD group.

	ASD
AQ Communication	0.20 (0.40)
AQ Social skills	−0.31 (0.18)
AQ Attention switching	0.03 (0.89)
AQ Attention to detail	**0.46 (0.045)**
AQ Imagination	−0.32 (0.17)
AQ Total	0.14 (0.54)
EQ Total	−0.10 (0.64)
SQ Total	**0.42 (0.04)**
General IQ	0.10 (0.57)
Verbal IQ	0.12 (0.51)

^a^ Data are presented as Spearman’s correlation coefficient (*p*-value). Statistically significant correlations are shown in bold (*p* < 0.05); ^b^ AQ: Autism spectrum quotient; ^c^ EQ: Empathy quotient; ^d^ SQ: Systemizing quotient.

## Data Availability

The data presented in this study are available on request from the corresponding author. The data are not publicly available due to their containing information that could compromise the privacy of research participants.
